# The Application of Pressure by the Tampon to the Cavity of the Uterus in Disease of the Endometrium

**Published:** 1886-07

**Authors:** V. H. Taliaferro

**Affiliations:** Atlanta, Georgia


### THE APPLICATION OF PRESSURE BY THE TAMPON TO THE CAVITY OF THE UTERUS IN DISEASES OF THE ENDOMETRIUM.

---

*To the Editors of the Atlanta Medical and Surgical Journal:*

At the late meeting of the State Medical Association, held at Augusta, Georgia, I made a report upon "The Application of Pressure, by Tampon, to the Cavity of the Uterus in Diseases of the Endometrium." In the interesting discussion by Drs. Campbell and Nunn which followed, you made the statement that a translation from the French, by Dr. V. O. Hardon, upon the same subject, would appear in the forthcoming issue of your journal. Since the publication of this number (June), I find the interesting translation alluded to, which is upon *Dilatation* of the Uterine Cavity by the Tampon. My report, as you will remember, treats of the tampon to the uterine cavity solely as a *curative* or *therapeutic* measure. In the one report the tampon is used as a mechanical means of dilatation, for purposes of diagnosis, while in the other it is used as a means of cure in the inveterate disease of the cavity of the uterus.

The subject of Dr. Hardon's translation is, "Vulliet's New Method of Dilating the Uterine Cavity." The subject of my paper is, "The Application of Pressure, by the Tampon, to the Cavity of the Uterus in Diseases of the Endometrium."

Until your announcement at the recent meeting of the State Association, I had never heard of Vulliet's new method of dilatation. Since my earliest experience with the tampon to the uterine cavity as a curative measure (now some two years ago), I have known of its effective powers of dilatation, but I had not attributed special importance to it as a means of diagnosis, for the reason that the dilatation is slow, and the pressure required to get thorough dilatation so rapidly modifies and changes the morbid structures that any opinion founded upon sight or touch, following such slow dilatation and pressure, would be merely conjectural.

The display of the fully dilated and exposed uterine surface is beautiful, but to get such a display of surface requires an amount of slow pressure as oftentimes to destroy the endometritis, mucus fungosities, polypoid growths, etc., that we are in search of.

It will be seen by my paper to the State Association, and by a more elaborate report to appear in the August issue of your journal, that the methods of tamponing the uterine cavity, both of Vulliet's and myself, are quite similar, however widely dissimilar the objects to be attained. Very truly yours,

V. H. TALIAFERRO, M. D.

Atlanta, Georgia, June 4, 1886.

---

A VALUABLE REMEDY FOR DIARRHŒA AND DYSENTERY.—The intestinal catarrhs so prevalent during the summer months make up a large proportion of the practice of every physician. While the treatment of this class of affections must necessarily be modified to suit the stage of the inflammation and the idiosyncrasies of the individual case, there are nevertheless certain symptoms common to all cases which may generally be relieved by a remedy which has been extensively used in Europe and in this country with the most gratifying success. We allude to chloranodyne, manufactured by Parke, Davis & Co. Chlor-anodyne is an improvement upon the proprietary preparation which, under the name of chlorodyne, was introduced into England by Dr. J. Collis Browne, and was widely used and commended by physicians of the highest standing. Chlor-anodyne is a happy combination of anodynes, anti-spasmodics and carminatives. It is diaphoretic, anodyne and astringent in its action, and, intelligently administered, has almost a specific action in diarrhœa and dysentery.—*Mississippi Valley Medical Monthly.*

